# Anabolic Steroids and Cardiovascular Outcomes: The Controversy

**DOI:** 10.7759/cureus.9333

**Published:** 2020-07-22

**Authors:** Jamal C Perry, Tayná M Schuetz, Mohammad D Memon, Sadaf Faiz, Ivan Cancarevic

**Affiliations:** 1 Medicine, California Institute of Behavioral Neurosciences & Psychology, Fairfield, USA; 2 Internal Medicine, California Institute of Behavioral Neurosciences & Psychology, Fairfield, USA

**Keywords:** anabolic steroids, cardiovascular

## Abstract

Anabolic steroids (AS) are synthetic derivatives of the male sex hormone testosterone. The use of AS is not limited to bodybuilders and athletes, but non-athletes also use them. It is used to enhance athletic performance, induce muscle hypertrophy, and augment male sexual characteristics. AS use is associated with a wide range of side effects and potential cardiovascular complications. In this article, we have searched the available literature to investigate the association between AS use and cardiovascular disease (CVD). The results revealed that AS was linked to lipid metabolism derangements, hypertension, coagulation disorders, and cardiomyopathy. We concluded, based on the relevant data, that there was evidence that suggests an association with CVD, primarily myocardial infarction, fatal arrhythmias, and cardiomyopathy in AS users. The general population should be informed of the risk. Also, methods of primary and secondary prevention should be implemented to mitigate the risk of CVD secondary to AS.

## Introduction and background

"Get strong or die trying" by powerlifter Ariel Stephens.

The sports industry has always been massive as it offers both the fame and financial status to athletes worldwide. In 1954, Olympics synthetic testosterone was first used by a Russian weightlifter, and subsequently, it became popular within the general population [1]. The World Anti-Doping Agency (WADA) estimates that 1% to 2% of athletes' urine samples test positive for performance-enhancing drugs [2]. A further 14% prevalence rate was observed by the use of biological passport measures. Also, in one anonymous survey of 2,167 world-class amateur athletes, about 43.6% admitted to using performance-enhancing drugs. It is estimated that 3.3% of teenagers in high school also use steroids [3]. Anabolic steroids (AS) are also used by non-athletes in order to improve their physical abilities and appearance [4].

AS are synthetic derivatives of the male sex hormone testosterone [5]. The correct term for these compounds is anabolic-androgenic steroids. "Anabolic" refers to muscle hypertrophy and "androgenic" refers to increased male sex characteristics. Drugs that are commonly used are testosterone, androstenedione, stanozolol (Winstrol), nandrolone (Deca-Durabolin), and methandrostenolone (Dianabol). Multiple research articles have shown that these drugs have a wide range of side effects, resulting in reproductive and metabolic disorders, psychological disturbances, cardiovascular disease (CVD), and renal and hepatic pathologies [6,7]. In this article, our key focus is on CVD. CVD encompasses a range of illnesses that include numerous pathologies of the heart and blood vessels. These include but are not limited to coronary artery disease, congestive heart failure, and arrhythmias. Heart disease is the number one cause of death globally [8]. The World Health Organization (WHO) estimated 17.9 million deaths by CVD in 2016, representing 31% of all global deaths. Of these deaths, 85% were due to heart attack and stroke.

The data on the association between AS and long-term cardiovascular morbidity and mortality are limited. As such, in this article, we will be searching the available literature with the PubMed database to investigate the association between AS use and CVD development due to the high prevalence of AS use.

## Review

_Hypertension and dyslipidemia_

AS have been associated with a range of adverse effects [9]. It is speculated that AS may be related to cardiovascular risk. Angell et al. suggested that there is no substantial data to confirm that AS is linked to hypertension and lipid profile alterations [10]. Moreover, the precise mechanism of cardiac effects remains a mystery. Corona et al. conducted a meta-analysis, which involved a randomized clinical trial of 3,016 AS users and 2,448 persons in a placebo group over a period of 34 weeks [11]. The analysis showed no metabolic derangements and suggested it might be cardioprotective because it reduced body fat and improved lean muscle mass among participants.

Vanberg and Atar posited that AS use was linked to disorders in lipid metabolism, elevations in blood pressure, and a procoagulant state overall, leading to CVD [12]. In 2010, Achar et al. highlighted that AS use could possibly result in the decrease of high-density lipoprotein (HDL) and an increase of low-density lipoprotein (LDL) among users, and they also linked it to elevations in blood pressure [13]. In 2015, Gheshlaghi et al. found that AS use was associated with a significant increase in both systolic and diastolic blood pressures, which positively correlates with drug duration [14]. Also, they found a decrease in LDL and no changes in HDL levels in plasma. However, this study was limited to a short period of two months among a small group of 267 athletes. In 2019, three studies supported the claim that AS use was related to dyslipidemia [15-17]. Rosca et al. conducted a study in rats and affirmed an increase in triglycerides and LDL with a decrease in HDL in plasma [15]. Souza et al. highlighted the mechanism of AS use, and stated it impairs HDL leading to the decrease in efflux of cholesterol, and may lead to early coronary artery disease [16]. Liu and Wu reported that AS use correlates with an increase of LDL in serum and decreases HDL levels in serum [17]. They further observed hypertension and accelerated atherosclerosis in combination, resulting in cardiovascular risk. 

**Figure 1:**
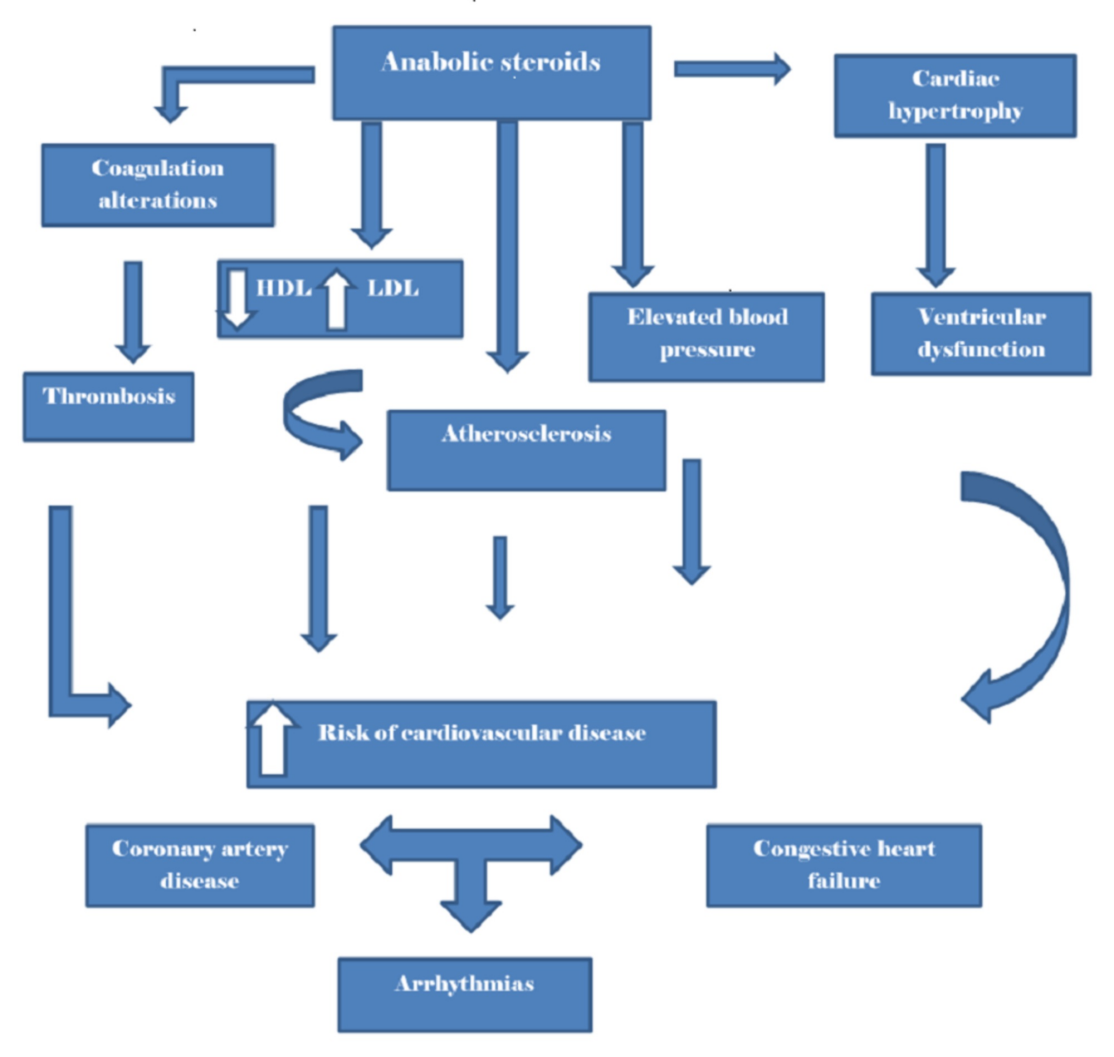
Anabolic steroids and cardiovascular outcomes This illustration depicts the relationship between anabolic steroid and cardiovascular disease. HDL, high-density lipoprotein; LDL, low-density lipoprotein.

We reviewed nine articles published between 2005 and 2019, investigating the relationship between AS use and CVD risk factors. Corona et al. found no cardiovascular risk; however, this study was conducted over a limited period and remains unclear about the accountability of high drug dose variations [11]. In contrast, some studies suggest the risk might be linked to chronic use, while others discovered drug effects in a short period of time. Overall, seven studies showed evidence of elevated blood pressure, alterations in lipid metabolism, and coronary atherosclerosis among AS users. Therefore, it can be concluded based on the majority of recent studies that AS is linked to CVD risk factors (Figure 1). It remains unclear the roles of AS drug dose and drug duration as it relates to CVD risk. As such, further research is needed to confirm this association.

_Cardiomyopathy and AS_

Li et al. reported a case of a healthy 22-year-old male bodybuilder that developed acute hepatic injury and rapidly progressive dilated cardiomyopathy after using stanozolol for 10 days [18]. This raises the question of how many younger athletes are at risk. Ha et al. reported a case involving an AS user who developed left ventricular hypertrophy after 20 years of AS use [19]. Pirompol et al. discovered cardiomegaly in rats over an 8-12 week period of using AS [20]. This was due to the suppression of myofilaments and the deposition of myocardial collagen. Søndergaard et al. analyzed the outcomes of congested heart failure due to AS use and concluded that AS-induced cardiomyopathy was non-reversible, and in the long term, patients may require cardiac devices or implantation [21]. Seara et al. also found that chronic AS use was linked to cardiac hypertrophy and myocardial ischemia due to a decrease in catalase mRNA expression [22]. In 2018, Barbosa Neto et al. concluded that AS users developed an increase in sympathetic modulation and high blood pressure, which were associated with alterations in the cardiac dimensions; this was primarily reflected in interventricular septal thickness and left ventricle posterior wall thickness [23]. 

**Figure 2:**
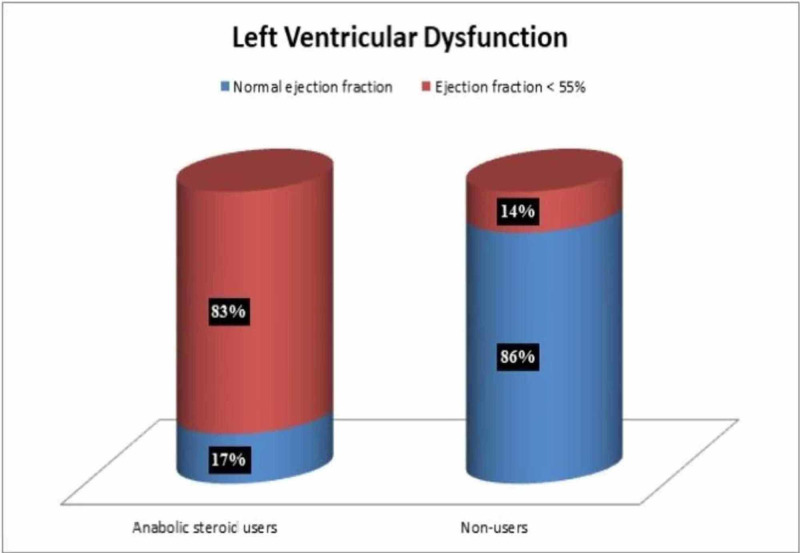
Left ventricular dysfunction The data represent 19 weightlifters of which 12 were anabolic steroid users, and seven were non-users, observed over 468 weeks.

There are several reports of individuals dying of CVD after the intake of AS in both athletes and non-athletes [24-27]. Far et al. found cardiac hypertrophy in 88 deceased males who tested positive for AS use [28]. Also, Montisci et al. found left ventricular hypertrophy in four autopsies and even an association with fibrosis, myocytolysis, and drug-induced eosinophilic myocarditis [29]. Cecchi et al. discovered a direct apoptotic cardiac and endothelial change in the heart tissue of deceased patients with heart failure who had a history of AS abuse [30]. In 2003, Hartgens et al. conducted two echocardiographic studies over a period of 8-16 weeks in 17 AS users and 15 non-users [31]. They found neither cardiac hypertrophy nor decreased function with AS use and argued that echocardiography may lack sensitivity in detecting adverse effects. Also, Nottin et al. found no increase in left ventricular wall thickness but reported decreased left ventricular function in AS users [32]. Baggish et al. reported findings of echocardiographic cardiac dysfunction among 83% chronic AS users in their study (Figure 2) [33]. Golestani et al. proposed the use of molecular imaging as a method for early detection for CVD in AS users [34]. Baumann et al. reported myocardial scaring and cardiomegaly, diagnosed by MRI in a male bodybuilder with 20 years of AS use [35]. Alizade et al. found an increase in right ventricular heart strain with the use of a two‐dimensional speckle tracking echocardiography [36]. On the other hand, D'Andrea et al. found left atrial dysfunction with the use of speckle echocardiography in AS users [37]. Rasmussen et al. reported an association between global left ventricular strain and AS use, which was detected by echocardiography and MRI [38]. 

We reviewed 21 articles published between 2003 and 2019 to determine if AS was linked to cardiomegaly and ventricular dysfunction. Collectively, 19 studies showed evidence that AS users developed cardiomegaly and ventricular dysfunction, and these findings were based on molecular imaging, echocardiography, or pathology reports (Figure 1). Moreover, it was commonly associated with long-term use. Nevertheless, most of these studies were limited by their sample size. Only a few suggested the actual mechanism of insult on the heart. Hence, further research is needed.

_Sudden cardiac death and AS_

The suspicion exists that there is a strong correlation between AS use and sudden cardiac death due to arrhythmias and myocardial infarction. In 1991, Ferenchick et al. suspected that there might be an association between AS and thrombosis that may lead to myocardial infarction and stroke in athletes [39]. Also, Shamloul et al. reported a case of a 37-year-old male AS user who died of ischemic stroke and myocardial infarction [27]. In 2011, Lippi and Banfi found that AS usage lead to thrombotic complications in athletes [40]. Roşca et al. also tested this theory with the drug nandrolone, and observed a hypercoagulable state in rats [41]. Chang et al. suggested that AS use may impair synthesis of coagulation factors, inhibitors, and fibrinolytic proteins, causing a procoagulant state that may lead to myocardial infarction and other thrombotic complications [42]. Baggish et al. also found that there was an increase in coronary artery plaque volume in AS users when compared to non-user, leading to rapidly progressive coronary artery disease [43].

Several studies revealed that AS use is linked to arrhythmia and may cause sudden cardiac death [44,45]. Two studies in 2015 and 2016, respectively, also affirmed an association with life-threatening ventricular arrhythmias in rats with the use of nandrolone [46]. Alizade et al. suggested that Tp-e interval, Tp-e/QT ratio, and Tp-e/QTc ratio were increased in bodybuilders that used AS, and this may lead to ventricular arrhythmias [47]. This was referenced in a case report published by Lichtenfeld et al. of an AS user who died of ventricular fibrillation [48]. In 2019, Marocolo et al. concluded that AS is associated with cardiac autonomic dysfunction and ventricular repolarization, and reflected an increase in QT interval [49].

We reviewed several articles published between 1991 and 2019 to determine if AS may lead to sudden cardiac death. Six studies indicated that AS might impair coagulation, leading to thrombotic complications and myocardial infarction. Another six studies linked AS to potentially life-threatening arrhythmias. Therefore, it can be deduced that AS is linked to CVD and may lead to sudden cardiac death (Figure 1). However, these studies were limited by their small sample size. We found only a few case reports that suggested AS was linked to sudden cardiac death. Further research is still needed.

_Limitations_

This study included animal studies and articles that were older than 10 years. It also lacked large-scale epidemiological studies; the majority of studies were based on a small population.

## Conclusions

In this article, we reviewed the available literature to investigate the association between AS use and CVD. We deduced that AS use was associated with CVD, in particular congestive heart failure, cardiac arrhythmias, and coronary artery disease. Minimal evidence against this association was found. Potential confounders would be polysubstance abuse, high caloric diet, and genetic factors. In summary, the individuals using AS developed CVD risk factors, such as elevated blood pressure and dyslipidemia. Also, they were at risk of developing cardiomyopathy and coagulation alteration. However, additional investigation is required to confirm AS use as a primary risk factor and to determine an actual census of individuals who may be at risk. Surveillance of AS users and efforts aimed at increasing awareness of the general population should also be recommended.
